# High expression of *IL4I1* is correlated with poor prognosis and immune infiltration in thyroid cancer

**DOI:** 10.1186/s12902-023-01407-1

**Published:** 2023-07-11

**Authors:** Liying Zhu, Jun Wang, Jia’an Hu

**Affiliations:** 1 Department of Geratology, Ruijin Hospital, Shanghai Jiao Tong University School of Medicine, No.197 Ruijin 2nd Road, Shanghai, 200025 China; 2 Department of Otolaryngology, Ruijin Hospital, Shanghai Jiao Tong University School of Medicine, Shanghai, China

**Keywords:** Thyroid cancer, *IL4I1*, Biomarker, Poor prognosis, Immune infiltration

## Abstract

**Background:**

Thyroid cancer-related deaths mostly result from metastasis. It was reported that the immunometabolism associated enzyme interleukin-4-induced-1 (IL4I1) was related to tumor metastasis. The present study was intended to investigate the effects of *IL4I1* on thyroid cancer metastasis and its relationship with the prognosis.

**Methods:**

Data from Cancer Genome Atlas (TCGA) and Gene Expression Omnibus (GEO) were analyzed to find out the different mRNA expressions of *IL4I1* between thyroid cancer and normal tissues. And Human Protein Atlas (HPA) was used to assess IL4I1 protein expression. To further differentiate thyroid cancer from normal tissues and estimate the impact of *IL4I1* on the prognosis, the receiver operating characteristic curve (ROC) and Kaplan–Meier (KM) method was performed. The protein–protein interaction (PPI) network was established using STRING, and functional enrichment analyses were conducted by “clusterProfiler” package. Then, we assayed the correlation between *IL4I1* and some related molecules. The relationship between *IL4I1* and immune infiltration was performed using “Gene Set Variation Analysis (GSVA)” package in TCGA and tumor-immune system interaction database (TISIDB). Finally, we did in vitro experiments in order to further prove the bioeffects of *IL4I1* on metastasis.

**Results:**

The expression of *IL4I1* mRNA and IL4I1 protein was significantly upregulated in thyroid cancer tissues. The increment of *IL4I1* mRNA expression was related to high-grade malignancy, lymph node metastases and extrathyroidal extension. The ROC curve displayed the cutoff value of 0.782, with the sensitivity of 77.5% and the specificity of 77.8%. KM survival analysis showed that there was a worse PFS in patients with high *IL4I1* expression than those with low *IL4I1* expression (*p* = 0.013). Further study indicated that *IL4I1* was associated with lactate, body fluid secretion, positive regulation of T cell differentiation, and cellular response to nutrients in Gene Ontology (GO) analysis. Moreover, *IL4I1* was found correlated with immune infiltration. Finally, the in vitro experiments revealed the promotion of *IL4I1* on cancer cell proliferation, migration and invasion.

**Conclusions:**

The increased *IL4I1* expression is markedly correlated with the immune imbalance in the tumor microenvironment (TME) and predicts poor survival in thyroid cancer. This study reveals the potential clinical biomarker of poor prognosis and the target of immune therapy in thyroid cancer.

**Supplementary Information:**

The online version contains supplementary material available at 10.1186/s12902-023-01407-1.

## Introduction

In the endocrine system, thyroid cancer is the most common type of endocrine tumors, accounting for approximately 1.7% of all human malignancies [1, 2]. Based on histopathological features, thyroid cancer can be divided into papillary thyroid cancer (PTC), anaplastic thyroid cancer (ATC), follicular thyroid cancer (FTC), and medullary thyroid cancer, of which PTC is the most common type [3]. Most PTC is well-differentiated and has a good prognosis with the mortality rates of 3–10%, but the mortality rates of poorly-differentiated PTC (PDPTC) and ATC are 38–57% and ~ 100%, respectively [4]. Therefore, it is important to detect the thyroid cancer with unfavorable prognosis. Recently, active surveillance is recommended for thyroid cancer [5]. However, we still lack the diagnostic biomarkers and prognostic indices for the mechanisms underlying thyroid cancer metastasis have not been fully understood. Hence, it is pivotal to explore the molecular mechanism that drive metastasis and choose the available biomarkers for tumor surveillance and prognostic assessments in thyroid cancer.

Interleukin-4-induced-1 (IL4I1) is a secreted L-amino acid oxidase that oxidizes l-phenylalanine to corresponding α-ketoacids, H_2_O_2_, and ammonia [6, 7]. IL4I1 is reported expressed in macrophages, mature dendritic cells (DC), T cells, and B cells stimulated by interleukin (IL) -4. IL4I1 secreted by antigen presenting cells inhibits human CD4^+^ and CD8^+^ T lymphocyte activation and proliferation, in part via the production of H_2_O_2_ and some other intracellular signal pathways inside T lymphocytes [8, 9]. Besides, IL4I1 modulates B cell differentiation, especially those involved in the T-cell-dependent immune response [10–12]. Amélie et al. [13, 14] found that IL4I1 was expressed in tumor-associated macrophages (TAM) of most human malignancies, but only in rare solid tumor cells including mesotheliomas, non-small-cell carcinomas, thyroid carcinoma and ovarian carcinoma. As an immunosuppressive enzyme, IL4I1 plays an important role in tumor immune escape and predicts poor prognosis. Thus, IL4I1 could explain partially the mechanisms underlying tumor metastasis and be a potential diagnostic and prognostic biomarker of thyroid cancer.

Here, we intended to explore the correlation between IL4I1 and immune escape in thyroid cancer. In the first place, we evaluated the *IL4I1* mRNA and IL4I1 protein expression, and then analyzed its relationship with clinical characteristics (criterion of AJCC 7th) using data from Cancer Genome Atlas (TCGA), Gene Expression Omnibus (GEO) and Human Protein Atlas (HPA). Moreover, given the overexpression of *IL4I1* in thyroid cancer, especially in ATC, we hypothesized that *IL4I1* predicted poor survival in thyroid cancer. We analyzed data from TCGA and found that *IL4I1* was positively correlated to metastasis, poor prognosis and more immune infiltrates in tumor microenvironment. At last, we did some *in-vitro* experiments to verify *IL4I1* promotion of cancer proliferation and metastasis. Here, this study reveals that the high level of *IL4I1* promoted immune escape and predicted poor prognosis in thyroid cancer, which provides direction for future research into the novel target for immunotherapy, and diagnostic and unfavorable prognostic biomarker.

## Methods

### TCGA, and GSE50901, GSE27155 and GSE151181 datasets

TCGA database (https://genome-cancer.ucsc.edu/) [15] was used to analyze the *IL4I1* gene expression and its association to the corresponding clinical information. There were 33 cancer types involved and the data was transformed from FPKM into TPM format and log2 conversion when necessary. The gene expression in thyroid cancer from TCGA comprised 502 thyroid cancer tissues and 58 peritumoral normal samples. The transcriptional expression was also analyzed in other three public databases, GSE50901 [16, 17], GSE27155 [18, 19] and GSE151181 [20]. GSE50901 was obtained from the GPL13607 Agilent-028004 SurePrint G3 Human GE 8 × 60 K Microarray, and we chose data of 3 tumor matched PTC and 3 non-tumor matched samples for analysis. GSE27155 was obtained from GPL96 [HG-U133A] Affymetrix Human Genome U133A Array, including 51 PTC, 4 ATC and 4 normal thyroid tissue samples. GSE151181 contains two parts, of which one is miRNA expression data from GPL21575, and the other is gene array from GPL23159. In the present study, only data on gene expression was processed, including 15 tumor samples from PTC before radioiodine (RAI) treatment, 11 tumor samples from PTC after RAI treatment and 6 normal thyroid tissue samples.

### Human Protein Atlas (HPA)

The Human Protein Atlas (HPA) (https://proteinatlas.org/) [21, 22] includes the protein level of human genes in normal tissues, as well as in tumor tissues. The IL4I1 protein level was compared between thyroid cancer tissues and normal thyroid tissues in HPA.

### Protein–Protein Interaction (PPI) network construction

The online STRING database (http://string-db.org) [23] was adopted to retrieve the genes that interact with *IL4I1* gene, selecting the interaction score > 0.4 in the PPI network.

### Tumor-Immune System Interaction Database (TISIDB) web

The online web TISIDB [24] (http://cis.hku.hk/TISIDB/) integrates a repository portal for tumor immune system interaction. We analyzed the *IL4I1* expression and tumor-infiltrating immuocytes across human cancers using TISIDB. Besides, the correlations of *IL4I1* with some tumor-infiltrating lymphocytes (TILs) were measured using the *Spearman* test.

### Cell culture

Cell lines 8505C, TPC-1, KTC-1 and K1 [25] were given by the Department of Nuclear Medicine, Ruijin Hospital, Shanghai Jiao Tong University School of Medicine. All cell lines were cultured at 37 °C in a humidified atmosphere with 5% CO2 using RPMI 1640 (Invitrogen) with 10% fetal bovine serum (FBS; Gibco), 100 U/ml penicillin and 100 μg/ml streptomycin.

### Generation of 8505C sub-lines

Parental 8505C cells were infected with IL4I1shRNA-GFP-puro-lentivirus and GFP-puro-lentivirus in order to generate 8505C-shIL4I1 and 8505C-vector. After 72 h, 2 μg/ml puromycin was added to the transfected cells to obtain the population of anti-puro cells.

### Quantitative real-time polymerase chain reaction (qRT-PCR)

Total RNA of the cells was extracted using MolPure® Cell/Tissue Total RNA Kit (YEASEN, China). cDNA was synthesized using Hifair® III 1st Strand cDNA Synthesis SuperMix for qPCR (gDNA digester plus) (YEASEN, China). qPCR was performed using Hieff® qPCR SYBR Green Master Mix (High Rox Plus) (YEASEN, China) on ABI 7500. Primers sequences for the detection of *IL4I1* were 5’-CGCCCGAAGACATCTACCAG-3’ (forward) and 5’-GATATTCCAAGAGCGTGTG CC-3’ (reverse). The primer of *Gapdh* was 5’-GCAGGGGGGAGCCAAAAGGG-3’ (forward) and 5’- TGCCAGCCCCAGCGTCAAAG-3’ (reverse).

### Western blot

For immunoblot analyses, cells were lysed by cell lysis buffer for Western and IP containing protease inhibitor cocktail and quantified using Bradford Protein Quantification Kit (YEASEN, China). Protein was separated by SDS/PAGE, transferred to a PVDF membrane and probed with antibodies against IL4I1 (Abcepta, China) and GAPDH (Sangon Biotech, China).

### Cell proliferation assay

Cell counting kit-8 (CCK8) was used to evaluate the proliferation of the 8505C with *IL4I1* knocked down according to the manufacturer’s instruction (Sangon Biotech, China). In six 96-well plates, 1,000 cells per well were seeded. And 10 μl per well of CCK8 solution was added on day 1, day 2, day 3 and day 4 after seeding. Each plate was measured at 450 nm in a Multiscan MK3 plate reader (Thermo Fisher Scientific, Rochester, NY, USA).

### Migration and invasion assays

8505C-shIL4I1 and its control were starved in a serum-free medium overnight. The next day, all cells were plated at equal density in the serum-free medium into 8 μm pore size 24-well transwell migration or invasion inserts (Corning). 20% FBS medium was added to the bottom well and the plates were incubated at 37 °C in a humidified atmosphere with 5% CO2 for 24 h. The upper inserts were fixed and stained with crystal violet. The non-migrated cells were removed from the inserts. The pictures were taken under the light microscope. The migrated cells were washed with 33% acetic acid and the OD value was read at 560 nm in a Multiscan MK3 plate reader (Thermo Fisher Scientific, Rochester, NY, USA).

### Statistical analyses

R (V 3.6.3) was used for the statistical analysis. R package ClusterProfiler and ggplot2 were used to analyze functional enrichment and visualize expression differences. The statistical calculation was done by paired *t*-test and Mann–Whitney *U*-test. The survival curve was constructed using the KM method and the log-rank test. pROC package [26] was used to detect the cutoff of *IL4I1* in the ROC curve. Cox regression was applied to evaluate the hazard ratio (HR) for the progress free interval (PFI). Two-sided *t*-test was used where the data met the assumptions of the test and the variance was similar between the two groups being compared. GraphPad Prism 6 was used to analyze data. All *p* values of less than 0.05 were considered significant.

## Results

### Expression profile of *IL4I1* in pan-cancer and thyroid cancer

**Fig. 1:**
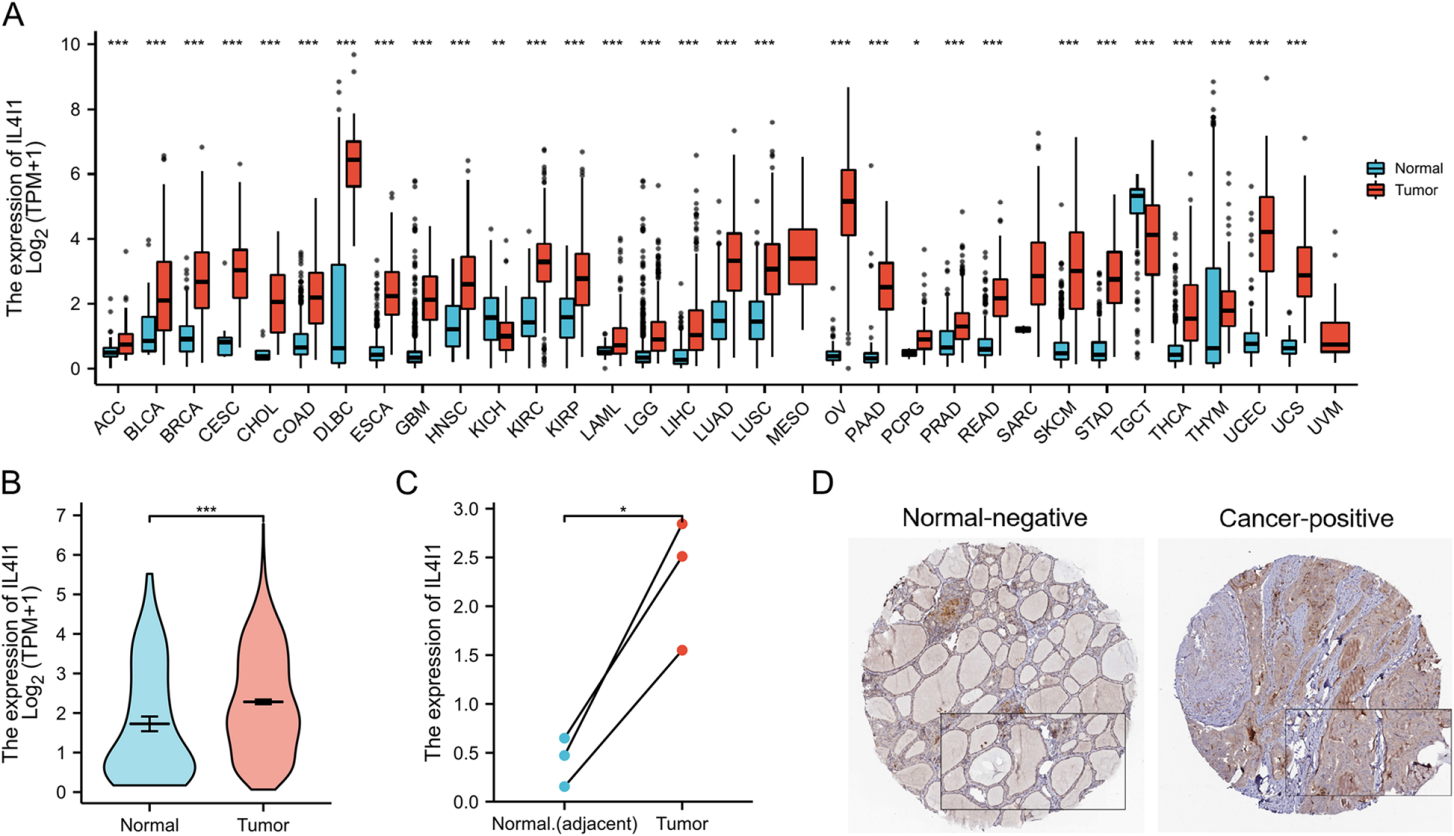
The pattern of IL4I expression in Pan-cancers and in thyroid cancer. **A** The mRNA expression of *IL4I1* was upregulated in 28 of 33 cancers (*** *p* < 0.001), in KICH (** *p* < 0.01) and in PCPG (* *p* < 0.05) compared with normal tissues. **B** The mRNA expression in THCA from data of TCGA. **C** The mRNA expression in 3 THCA and the matched-adjacent normal samples. **D** The protein levels of IL4I1 from data of Human Protein Atlas. ns, no significance; BLCA, bladder urothelial carcinoma; ACC, adrenocortical carcinoma; BLCA, bladder urothelial carcinoma; BRCA, breast invasive carcinoma; CESC, cervical squamous cell carcinoma and endocervical adenocarcinoma; CHOL, cholangiocarcinoma; COAD, colon adenocarcinoma; DLBC, lymphoid neoplasm diffuse large B-cell lymphoma; ESCA, esophageal carcinoma; GBM, glioblastoma multiforme; HNSC, head and neck squamous cell carcinoma; KICH, kidney chromophobe; KIRC, kidney renal clear cell carcinoma; KIRP, kidney renal papillary cell carcinoma; LAML, acute myeloid leukemia; LGG, brain lower grade glioma; LIHC, liver hepatocellular carcinoma; LUAD, lung adenocarcinoma; LUSC, lung squamous cell carcinoma; MESO, mesothelioma; OV, ovarian serous cystadenocarcinoma; PAAD, pancreatic adenocarcinoma; PCPG, pheochromocytoma and paraganglioma; PRAD, prostate adenocarcinoma; READ, rectum adenocarcinoma; SARC, sarcoma; SKCM, skin cutaneous melanoma; STAD, stomach adenocarcinoma; TGCT, testicular germ cell tumors; THCA, thyroid carcinoma; THYM, thymoma; UCEC, uterine corpus endometrial carcinoma; UCS, Uterine Carcinosarcoma; UVM, Uveal Melanoma

To estimate the gene expression profile of *IL4I1* in various cancer types, we included 33 types of cancers containing more than three samples in both the normal and the cancer groups. As is shown in Fig. 1A, *IL4I1* was expressed more in tumor groups than in the normal groups in 31 types of all 33 cancer types, including thyroid cancer (Fig. 1A and B). In the GSE50901 matched data, we obtained similar results that *IL4I1* expression was higher in tumor tissues (*n* = 3) than that in the adjacent normal tissues (*n* = 3), as shown in Fig. 1C (*p* < 0.05). There are seven immunohistochemistry (IHC) samples stained with IL4I1 antibody from 4 patients in HPA. One patient suffered from follicular adenoma carcinoma, and the other three patients are from papillary adenocarcinoma. The IHC also showed that IL4I1 protein expression was higher in cancer tissues in thyroid cancer (Fig. 1D). All these data indicated that cancer tissues had abnormally higher expression of *IL4I1* in both mRNA and protein levels in thyroid cancer.

### The relationship between high expression of *IL4I1* mRNA and poor clinicopathologic characteristics of thyroid cancer

**Fig. 2:**
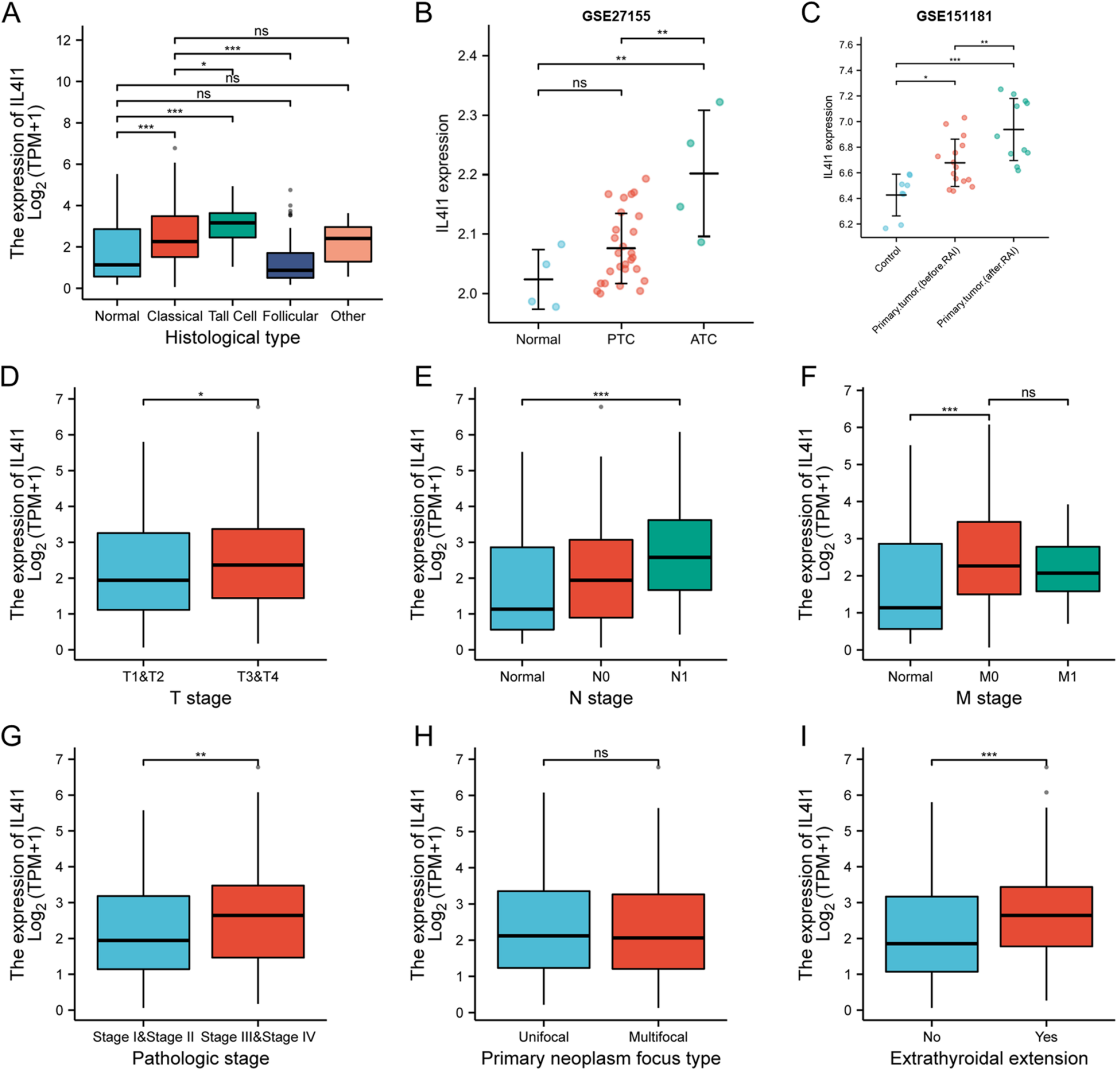
The relationship between *IL4I1* expression and clinical characteristics. The expression of *IL4I1* was statistically correlated with histological type (**A** and **B**), RAI (**C**), T stage (**D**), N stage (**E**), pathologic stage (**G**) and extrathyroidal extension (**I**). And there was no significant correlation between *IL4I1* expression and M stage (**F**) and primary neoplasm focus type (**H**). (ns, no significance, *p < 0.05, **p < 0.01, ***p < 0.001). PTC, papillary thyroid cancer; ATC, anaplastic thyroid cancer; RAI, radioiodine therapy

The correlation between *IL4I1* expression and clinical pathological features was evaluated by Mann–Whitney *U*-test and logistic regression analysis. TNM stages used here are according to AJCC 7^th^. From Fig. 2 and Table 1, we saw that higher expression of *IL4I1* is related to higher malignancy grades (Fig. 2A and B, *p* < 0.01), TNM stage (Fig. 2D, E and F, *p* < 0.05), and pathologic stage (Fig. 2G, *p* < 0.01) in thyroid cancer. Furthermore, patients with extrathyroidal metastasis had obvious higher *IL4I1* mRNA expression (Fig. 2I, *p* < 0.001). It is interesting that *IL4I1* was upregulated in thyroid cancer tissues (Fig. 2C, *p* < 0.05), and even higher after (RAI) in GSE151181 database (Fig. 2C, *p* < 0.01). The effects of RAI on *IL4I* expression were recently not clear.

**Table 1:** Clinical characteristics in thyroid cancer from TCGA data

Characteristic	Low expression of *IL4I1*	High expression of *IL4I1*	*p*
n	255	255	
T stage, n (%)			0.007^*^
T1	71 (14%)	72 (14.2%)	
T2	100 (19.7%)	67 (13.2%)	
T3	75 (14.8%)	100 (19.7%)	
T4	8 (1.6%)	15 (3%)	
N stage, n (%)			< 0.001^*^
N0	126 (27.4%)	103 (22.4%)	
N1	88 (19.1%)	143 (31.1%)	
M stage, n (%)			1.000
M0	129 (43.7%)	157 (53.2%)	
M1	4 (1.4%)	5 (1.7%)	
Age, meidan (IQR)	46 (34.5, 58)	46 (36, 58)	0.883

^*^p < 0.05

### Overexpression of *IL4I1* mRNA prediction of poor progress-free interval (PFI) in the TCGA database

**Fig. 3:**
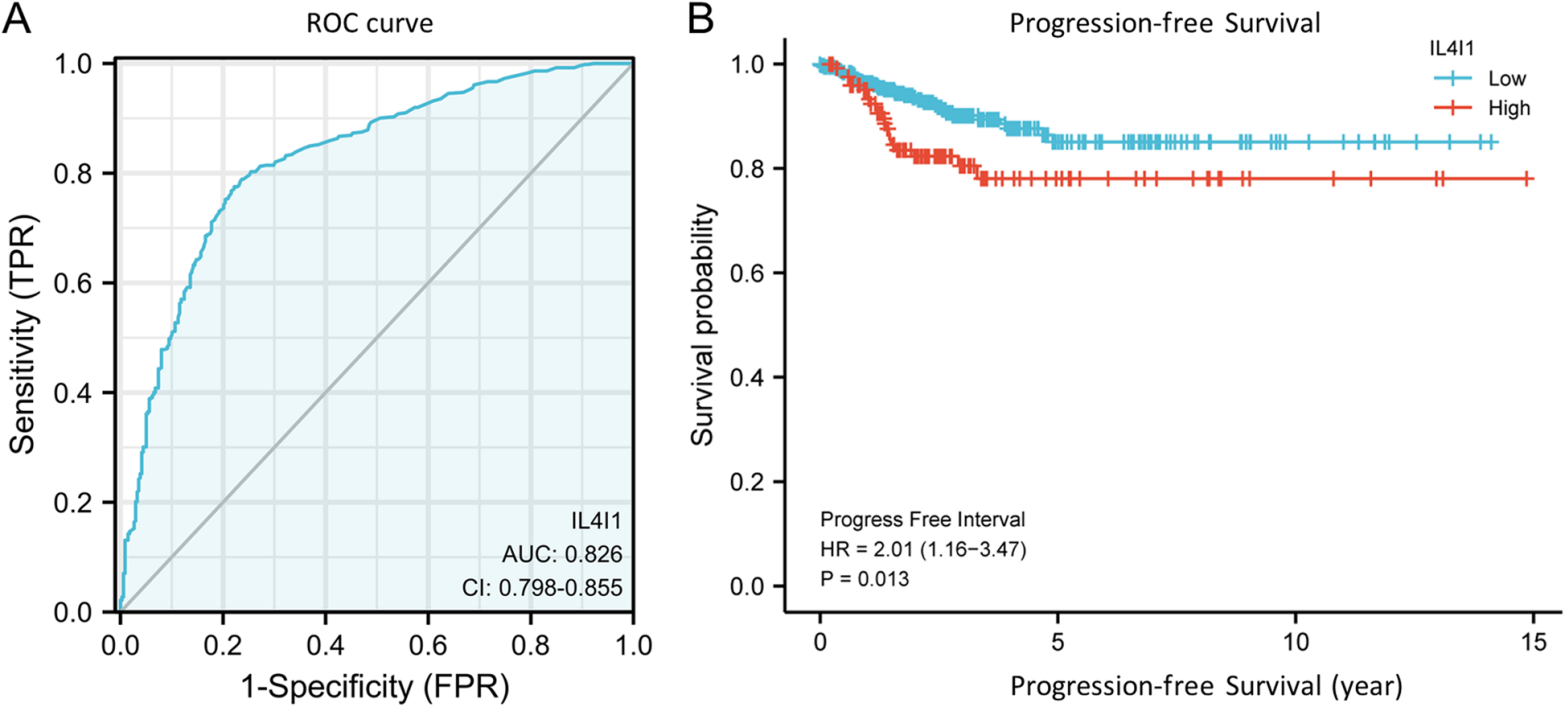
ROC and Kaplan–Meier curves for *IL4I1*. **A** ROC curve showed that IL4I1 had an AUC value of 0.826 to discriminate thyroid cancer tissues from normal controls with a cutoff of had no significant statistical difference between high *IL4I1* expression and low expression (*p* = 0.166) indicated by Kaplan–Meier survival curves. **B** Kaplan–Meier survival curves showed that thyroid cancer patients with high expression of *IL4I1* had shorter of PFS) (62 vs. 20 years, *p* = 0.013)

To explore the effects of *IL4I1* on the prognosis of thyroid cancer, we first performed the univariate Cox analysis. The results revealed that *IL4I1* was one of the risk factors in thyroid cancer based on the TCGA database (HR 1.894; 95% CI 1.083–3.310; *p* = 0.025; Table 2). Then, we performed ROC analysis and Fig. 3A displayed an AUC value of 0.826 (95% CI: 0.798–0.855). The cutoff value was 0.782, with a sensitivity of 77.5%, and a specificity of 77.8%. The positive predictive value was 84.1% and the negative predictive value was 69.6%. KM curves (Fig. 3B) showed that thyroid cancer patients with high-level *IL4I1* had a significantly poor prognosis (HR 2.01; 95% CI 1.16, 3.47; *p* = 0.013). These data referred that high *IL4I1* level would be a clinical biomarker indicating a poor prognosis of thyroid cancer.

**Table 2:** Univariate and multivariate Cox proportional hazards analysis of IL4I1 expression

Characteristics	Total(N)	Univariate analysis		Multivariate analysis	
		Hazard ratio (95% CI)	*P* value	Hazard ratio (95% CI)	*P* value
Gender (Male vs. Female)	510	1.694 (0.975–2.945)	0.062	1.390 (0.767–2.519)	0.278
Age (> 45 vs. < = 45)	510	1.593 (0.920–2.758)	0.096	1.558 (0.856–2.835)	0.147
T stage (T3&T4 vs. T1&T2)	508	2.450 (1.417–4.236)	0.001^*^	2.134 (0.925–4.925)	0.076
N stage (N1 vs. N0)	460	1.658 (0.936–2.934)	0.083	1.267 (0.685–2.344)	0.450
Extrathyroidal extension (Yes vs. No)	492	1.874 (1.092–3.216)	0.023^*^	0.867 (0.385–1.955)	0.731
Primary neoplasm focus type (Multifocal vs. Unifocal)	500	1.025 (0.595–1.764)	0.929	/	/
*IL4I1* (High vs. Low)	510	1.894 (1.083–3.310)	0.025^*^	1.568 (0.860–2.860)	0.142

^*^p < 0.05

### The interaction analysis of *IL4I1* with the related molecules

**Fig. 4:**
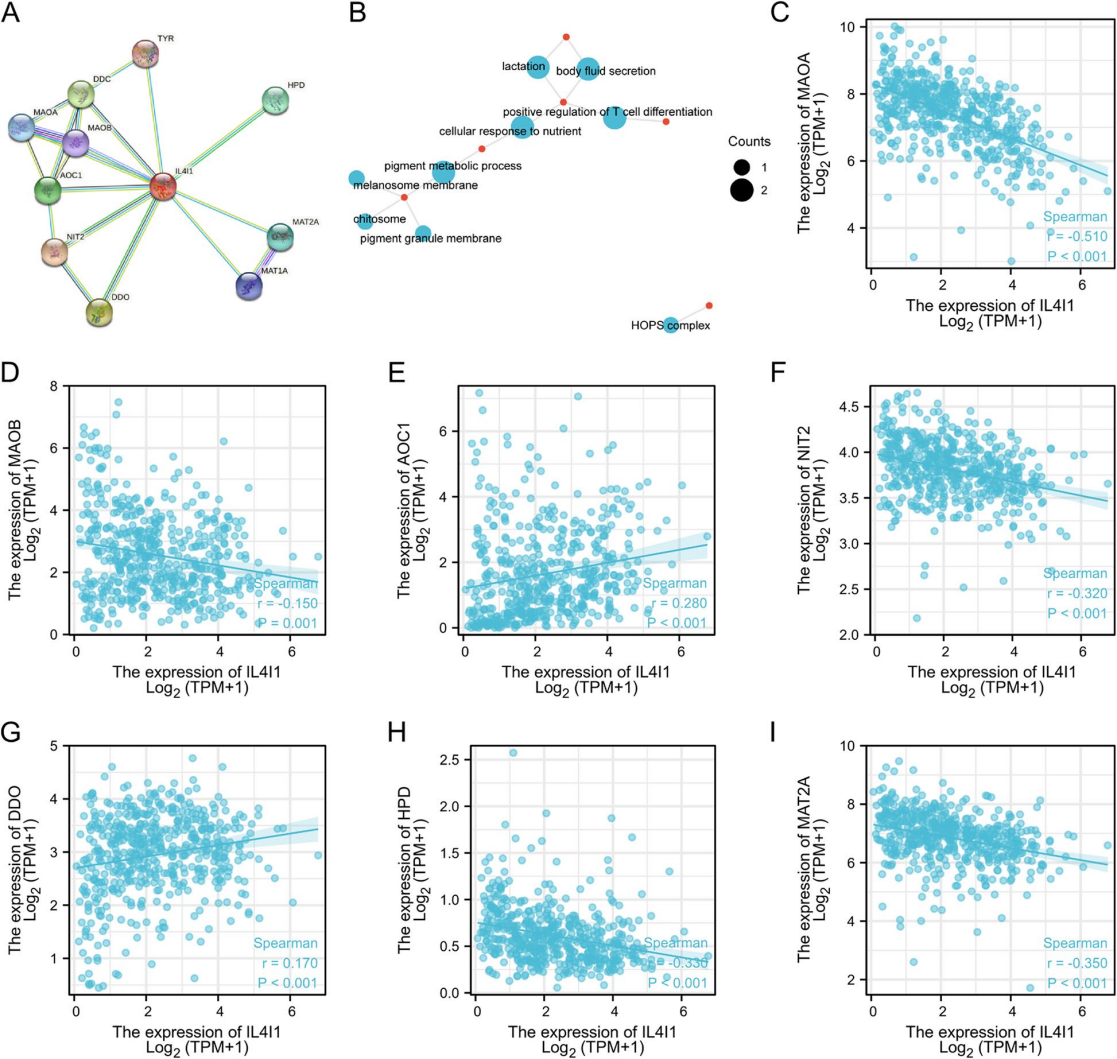
PPI network and functional analysis. **A** A network of IL4I1 and its co-expression genes. **B** Functional enrichment analysis of 11 involved genes. IL4I1 was involved in lactation, positive regulation of T cell differentiation, body fluid secretion, cellular response to nutrient, pigment metabolic process. **C-I** The correlation analysis between IL4I1 expression and co-expressed genes in thyroid cancer. MAOA, monoamine oxidase A; MAOB, monoamine oxidase B; AOC1, amine oxidase copper containing 1; NIT2, nitrilase family member 2; DDO, D-aspartate oxidase; HPD, 4-hydroxyphenylpyruvate dioxygenase; MAT2A, methionine adenosyltransferase 2A

STRING database [23] was used to build protein–protein interaction (PPI) networks. Figure 4A showed the network of *IL4I1* and its 10 co-expressed genes. Gene Ontology (GO) analysis revealed the biological process of *IL4I1*, including lactation, body fluid secretion, positive regulation of T cell differentiation, cellular response to nutrient and pigment metabolic process (Fig. 4B). The *Spearman* correlation analysis (Fig. 4C-I) demonstrated that there was negative relationship between *IL4I1* expression and MAOA (*r* = -0.51), MAOB (*r* = -0.15), NIT2 (*r* = -0.32), HPD (*r* = -0.33), MAT2A (*r* = -0.35), and there was positive relationship between *IL4I1* expression and AOC1 (*r* = 0.28,), DDO (*r* = 0.17) in the co-expressed genes in thyroid cancer from TCGA dataset (all *p* values ≤ 0.001).

### The correlation between *IL4I1* level and infiltrating immunocytes

**Fig. 5:**
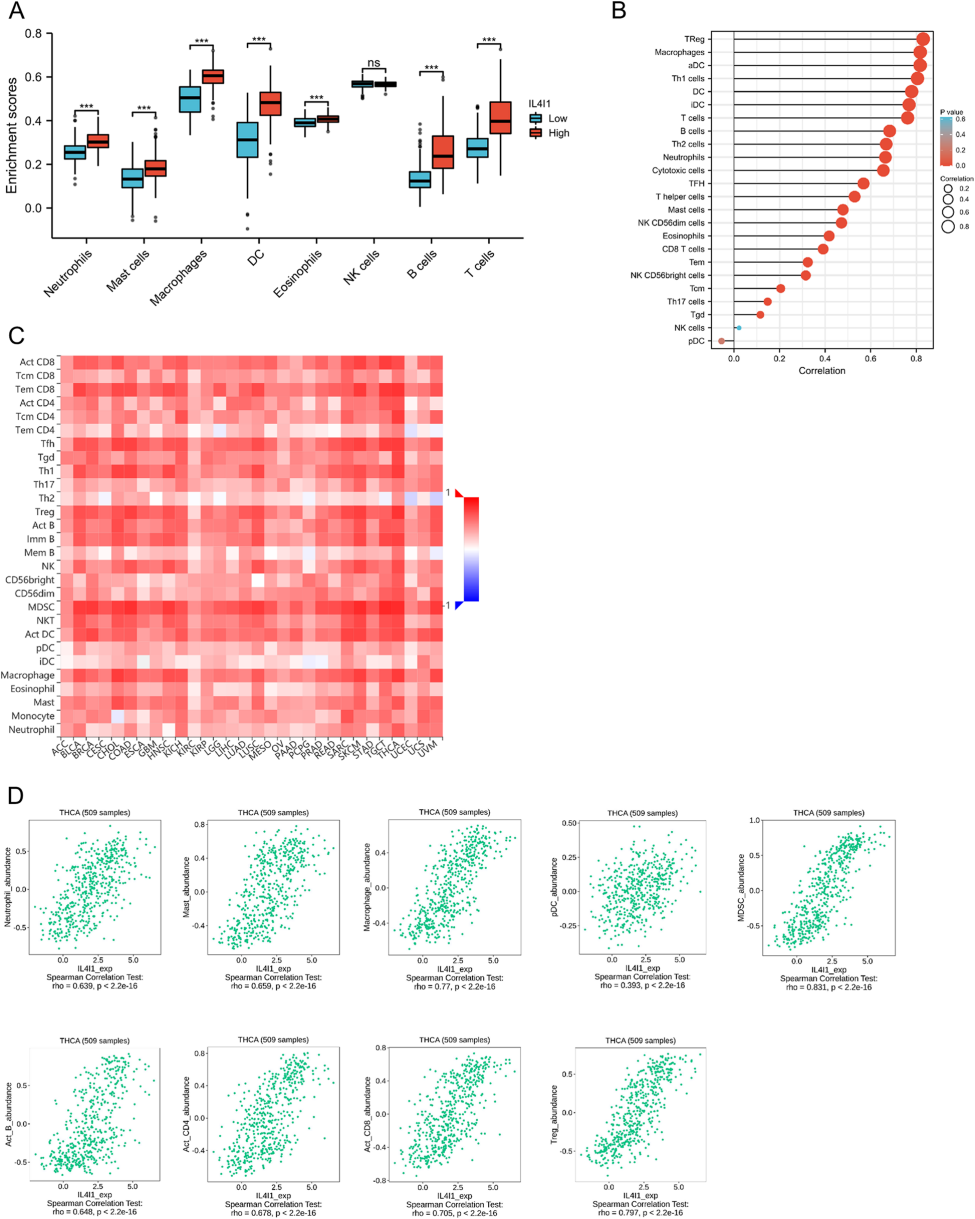
Correlation of *IL4I1* expression with immune cell infiltration level. **A** The high *IL4I1* expression is correlated macrophage, dendritic cells (DC), T cells, B cells and neutrophils. **B** The correlation analysis between *IL4I1* expression and the specific types of the immune cells mentioned above. **C** Relations between the *IL4I1* expression and 28 types of tumors infiltrating lymphocytes (TILs) across human cancers. **D** *IL4I1* expression was correlated with abundance of MDSC, Treg cells, macrophage, CD8 + T cells, CD4 + T cells, mast cells, B cells and neutrophil and DC cells

IL4I1 is an immune-associated enzyme secreted by tumor cells. Thus, we assayed the correlation of *IL4I1* expression with infiltrating immunocytes around the tumor cells to investigate the changes in the tumor immune microenvironment. In TCGA database, thyroid cancer with high expression of *IL4I1* mRNA had more infiltrating neutrophils, mast cells, macrophages, DC, eosinophils, B cells and T cells, but there was no difference in NK cells (Fig. 5A-B). *Spearman* correlation analysis revealed that *IL4I1* expression was significantly related to infiltrating T cells including Treg and Th1 cells (total T cells: *r* = 0.763; Tregs, *r* = 0.831; Th1, *r* = 0.806; all *p* < 0.001), DC including active DC (aDC) and interdigitating DC (iDC) (DC, *r* = 0.779; aDC, *r* = 0.817; iDC, *r* = 0.768; all *p* < 0.001) and macrophage (*r* = 0.818, *p* < 0.001). At last, we evaluated the correlation between *IL4I1* level and the abundance of 28 types of TILs in different human cancers in the TISIDB database, and the results was shown in Fig. 5C. From Fig. 5D, we saw that the high *IL4I1* level was correlated with more myeloid-derived suppressor cells (MDSC, *r* = 0.831), Tregs cells (*r* = 0.797), macrophage (*r* = 0.77), CD8^+^ T cells (*r* = 0.705), CD4^+^ T cells (*r* = 0.678), mast cells (*r* = 0.659), neutrophil (*r* = 0.639), B cells (*r* = 0.648), and plasmacytoid DC (pDC, *r* = 0.393) (all *p* < 2.2x10^–16^). All these data illustrated that *IL4I1* could play a particular part in immunocytes infiltration into the tumor microenvironment (TME) in thyroid cancer, which could provide a new perspective on immune escape in thyroid cancer.

### *IL4I1* promoting cell proliferation, migration and invasion of thyroid cancer cells in vitro

**Fig. 6:**
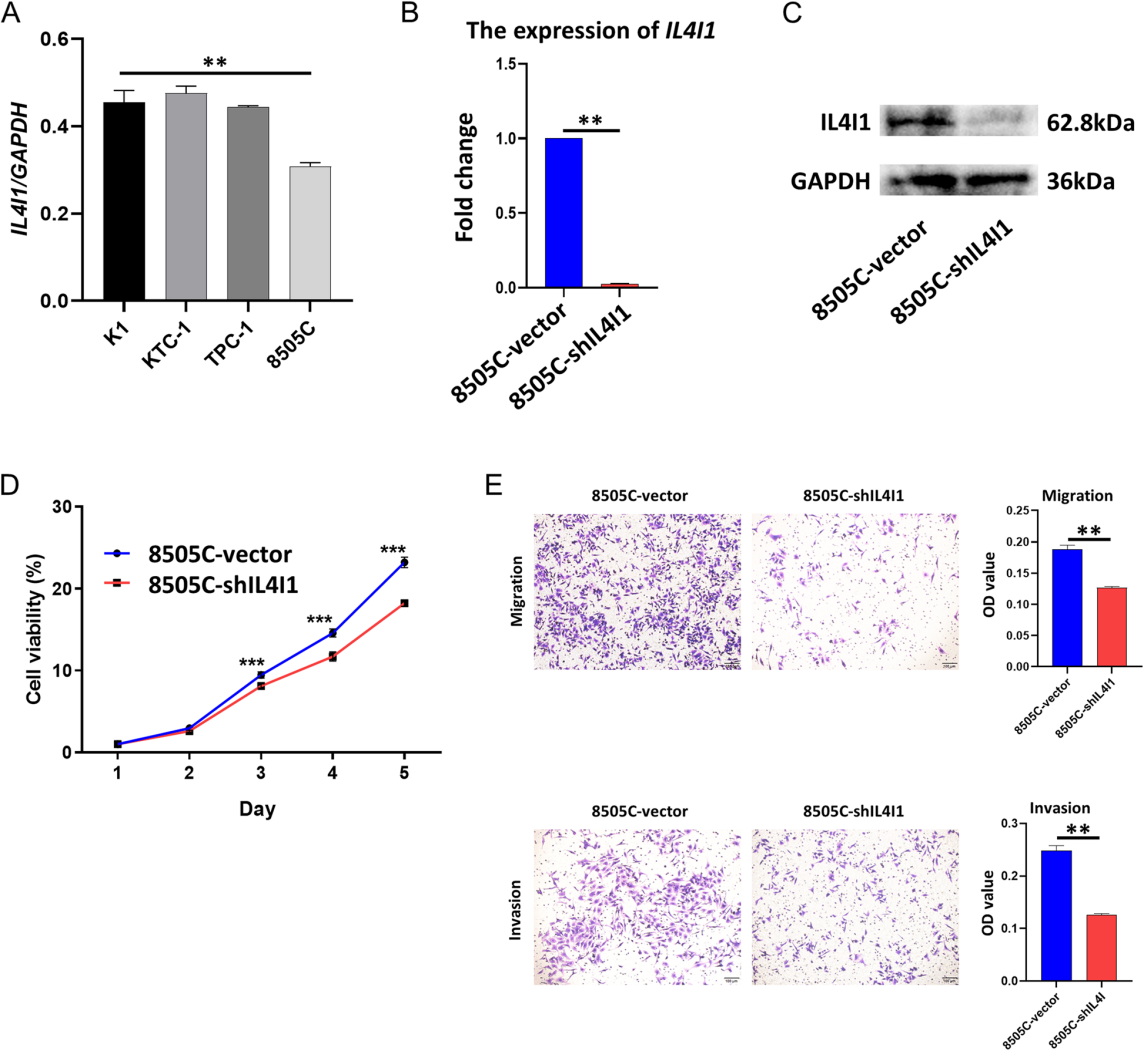
The effects of IL4I1 on cell proliferation and metastasis in *vitro*. **A** 8505C cell (ATC) had the highest *IL4I1* expression in the thyroid cancer cell lines K1, KTC-1, TPC-1 and 8505C tested by qPCR (** *p* < 0.01). **B** 8505C cell with IL4I1 knocked down by lentivirus containing shRNA targeting IL4I1 (8505C-shIL4I1) had lower *IL4I1* expression compared with its control (8505C-vector) (** *p* < 0.01). **C** 8505C-shIL4I1 cell had lower IL4I1 protein level compared with 8505C-vector (cropping blots). **D** The cell proliferation assay showed the impaired proliferation without IL4I1 (*** *p* < 0.001). **E** The migration and invasion ability of 8505C-shIL4I1 cell was significantly weaken (** *p* < 0.01)

Finally, we examined the *IL4I1* expression in four thyroid cancer cell lines (K1, KTC-1, TPC-1 and 8505C). Interestingly, a much higher *IL4I1* expression was found in the 8505C cells (Fig. 6A, *p* < 0.01). Moreover, we established stably transfected 8505C cell lines expressing shRNA targeting *IL4I1* (8505C-shIL4I1) and its control, 8505C-vector (Fig. 6B, C) to study the effects of *IL4I1* on cell proliferation and metastasis. The *IL4I1* knockdown reduced the in vitro proliferation (Fig. 6D), and decreased the number of 8505C cells migrating across the membrane and the matrixgel invasion (Fig. 6E). Collectively, these data suggest that *IL4I1* sustains the proliferation and metastasis potential of thyroid cancers cells in vitro.

## Discussion

The incidence of thyroid cancer has increased in the past 30 years, especially in some Asian countries, such as China. With the development of sensitive ultrasonography and computed tomography (CT) scan, we have faced more overdiagnosis for 4 ~ 11% of thyroid tumors are asymptomatic and only found in autopsy studies [27]. Though kinds of researches find the mutations in *BARF* gene, *RAS* gene and *TERT* gene are related to aggressiveness, these mutations may also lead to a potentially excessive treatments, such as more surgeries. Thus, it is necessary to understand the mechanism of thyroid cancer metastasis and to explore effective biomarkers to discriminate metastatic thyroid cancers with poor prognosis from benign ones. Immune escape is one of the pivotal mechanism contributing to malignancy [28]. Some immune checkpoint inhibitors, such as anti-CTLA-4 and anti-PD-L1/PD-1, have been demonstrated in helping to eliminate cancer cells [29, 30]. And some ongoing clinical trials found the therapeutic effects of immune treatment on advanced thyroid cancer [31, 32]. These studies prompted us to examine immune imbalance in aggressive thyroid cancer in depth. As an immune-associated enzyme, interleukin-4-induced-1 (IL4I1) was found overexpressed in tumor cells and to be a parameter predictive of poor prognostic in breast cancer and renal cancer [33]. However, little is known about the IL4I1 biology, including the enzymology, the substrate ranges, the role of downstream metabolites, and the association between IL4I1 and different cancers. Besides, there has been no research on the biological effects of IL4I1 on thyroid cancer. Hence, we conducted this study and found that IL4I1 expression is upregulated in thyroid cancer both in mRNA and in protein level. The upregulated *IL4I1* mRNA expression is positively related to lymph node metastasis, high TNM stage and extrathyroidal extension. And high *IL4I1* mRNA expression is associated with shorter PFS, which indicated that *IL4I1* could be a potential biomarker for poor prognosis in thyroid cancer. What’s more, *IL4I1* also predicted the specific immune infiltration in thyroid cancer. The in vitro experiments showed the high expression of *IL4I1*in the aggressive cell line, 8505C cells, and *IL4I1* promoted the proliferation, migration and invasion of 8505C cells.

IL4I1 was first identified in B lymphoblasts which were induced by treatment with IL-4 [34]. For a long time, it was recognized as an L-amino acid oxidase specifically in leukocytes, which oxidizes aromatic amino acids. IL4I1 protein localized in lysosomal, functioned in antigen processing in B cells, and thus act in the immune response. Also, IL4I1 could be secreted into the extracellular environment regulating the immune response. The *IL4I1* gene was believed to be expressed restricted to cells of the immune system previously [35]. Further studies found the expression of *IL4I1* in the testis and brain both in humans and mice, and there are five isoforms of *IL4I1* encoded by the human gene. Isoform 1 is expressed in lymphoid tissue and isoform 2 is expressed in the central nervous system and spermatozoa, while other isoforms are little known recently [36–38].

In this study, we found that *IL4I1* was highly expressed in thyroid cancer, and *IL4I1* was related to malignant tumor types, such as ATC in thyroid cancer and poor prognosis. In 2003, Copie-Bergman C, et al. [39] initially found the overexpression of IL4I1 in tumor tissues of primary mediastinal B-cell lymphoma. Then, a study on IL4I1 expression by immunochemistry in more than 30 types of human tumor tissues reported that IL4I1 expressed in almost all tumor-infiltrating macrophages, but only in specific types of cancers, such as mesothelioma and B cell lymphomas. Some researchers showed that the tumor tissues expressing IL4I1 biologically has a good prognosis with overexpression of IL4I1 [13]. On the contrary, IL4I1 was found suppressing the effective anti-tumor T-cell response in non-hematological tumors infiltrated by macrophages. As a result, the high expression of IL4I1 in tumor cells promoted the immune escape in human primary cutaneous melanoma [40]. Recent studies suggested that IL4I1 catalyzed tryptophan into indole-3-pyruvate (I3P) and promoted cancer cell motility and metastasis through. I3P-KynA/I3A metabolic pathway [41, 42]. Tiffany Horng [43] et al. found that *IL4I1* promoted tumor proliferation by local anti-ferroptosis pathways via its metabolite I3P. Our study found that *IL4I1* promoted the proliferation, migration and invasion of 8505C cells probably via immunometabolism of tryptophan catabolism.

The importance of *IL4I1* expression in thyroid cancer metastasis and prognosis promoted us to explore the possible potential mechanism. Until recently, it is found that there were many immune cells in the microenvironment of the thyroid cancer and the type and density of the tumor-infiltrating immune cells were correlated with the prognosis in thyroid cancer patients [44]. Around the thyroid cancer cells were many immunosuppressive cells such as MDSC, Tregs (regulatory T cells), macrophages, and so on. Besides, thyroid cancer cells upregulated negative immune checkpoints, such as CTLA-4 (cytotoxic T-lymphocyte associated protein 4) and PD-L1 (programmed death-ligand 1), as well as immunosuppressive enzymes (IDO1, indoleamine 2,3-dioxygenase 1). MDSC is a type of immune cells induced by the tumor that mediates immune tolerance. Angell et al. [45] found that MDSC could be used to detect malignancy and predict the extent of disease and risk of persistence of thyroid cancer. Aggressive features of thyroid cancer and the lymph node metastasis had a high density of Treg infiltration [46, 47]. TAMs are the typical immune cells in the microenvironment of thyroid cancer and their infiltration was related to larger tumors, lymph node metastasis, capsular invasion, extrathyroidal extension and poor prognosis [48–50]. CD8^+^ T cells were usually considered the anti-tumor immune cells, but the enrichment of CD8^+^ T cells was associated with thyroid cancer recurrence, because CD8^+^ cells around tumor cells were found at the state of anergy [51].

What’s more, immunoregulatory enzymes, such as IDO1 and IL4I1, also created and sustained the tumor-tolerant microenvironment, which is expressed either by the tumor cells or by immune cells [7, 52]. IDO1 and IL4I1 were both oxidoreductases catalyzing the degradation of tryptophan to kynurenine. It is reported that cancers expressed high levels of IL4I1, inducing the differentiation of Tregs, promoting CD8^+^ T cell death, recruiting immunosuppressive TAMs and suppressing T cell proliferation and function via the Kyn-AHR axis [42]. In this study, we found that *IL4I1* was positively related to infiltrating MDSC, Treg, macrophage, and CD8^+^ T cells, which implied that *IL4I1* promoted the metastasis of thyroid cancer by inhibiting the anti-tumor immune response.

We found and verified the high expression of *IL4I1* in thyroid cancer and explored the immune mechanism of its promotion in metastasis. Nevertheless, there are some limitations to this study. First, there were para-tumor tissues representing normal tissues in TCGA and there was a small sample size included in the normal group included in the analysis. Therefore, the bias was inevitable. Second, the microarray-based bioinformatic analysis is powerful in analyzing the molecular mechanisms and in verifying the effects of *IL4I1* as a potential biomarker of poor prognosis in thyroid cancer, but experiments may be needed to confirm the relationship and the regulation of *IL4I1* on immune cells in the tumor microenvironment. And it is interesting to perform further studies using in vitro and in vivo experiments, like flow cytometry, immunocytochemistry and conditional culture et al. on the detailed cell types and mechanisms.

## Conclusions

In conclusion, *IL4I1* contributed to tumor metastasis and poor prognosis of thyroid cancer integrating the analysis of TCGA and GSE datasets. As the L-amino acid oxidase, *IL4I1* modulated amino acid metabolism, which is the pivotal nutrient of many tumor-infiltrating immune cells. Therefore, *IL4I1* was considered to regulate the proliferation, differentiation and function of tumor cells by immune escape. Our results suggest that *IL4I1* may be a potential diagnostic and prognostic marker for metastatic thyroid cancer. And further experiments are needed to prove the immunoregulating effect of *IL4I1*.

## Supplementary Information

**Additional file 1.**

**Additional file 2.**

## Data Availability

The data generated and analyzed in the current study are available in GSE50901 (https://www.ncbi.nlm.nih.gov/geo/query/acc.cgi?acc=GSE50901), GSE27155 (https://www.ncbi.nlm.nih.gov/geo/query/acc.cgi?acc=GSE27155), GSE151181 (https://www.ncbi.nlm.nih.gov/geo/query/acc.cgi?acc=GSE151181), TCGA database (https://genome-cancer.ucsc.edu/), HPA (https://proteinatlas.org/), STRING database (http://string-db.org) and TISIDB (http://cis.hku.hk/TISIDB/).

## Abbreviations

aDC: Active dendritic cells
AOC1: Amine oxidase copper containing 1
ATC: Anaplastic thyroid cancer
CCK8: Cell counting kit-8
CT: Computed tomography
CTLA-4: Cytotoxic T-lymphocyte associated protein 4
DC: Dendritic cells
DDO: D-aspartate oxidase
FTC: Follicular thyroid cancer
GEO: Gene Expression Omnibus
GO: Gene Ontology
GSVA: Gene Set Variation Analysis
HPA: Human Protein Atlas
HPD: 4-Hydroxyphenylpyruvate dioxygenase
HR: Hazard ratio
iDC: Interdigitating DC
IDO1: Indoleamine 2,3-dioxygenase 1
IHC: Immunohistochemistry
IL4I1: Interleukin-4-induced-1
KM: Kaplan-Meier
MAOA: Monoamine oxidase A
MAOB: Monoamine oxidase B
MAT2A: Methionine adenosyltransferase 2A
MDSC: Myeloid-derived suppressor cells
NIT2: Nitrilase family member 2
PD-L1: Programmed death-ligand 1
PDTC: Poorly differentiated thyroid cancer
PFI: Progress free interval
PPI: Protein-protein interaction
PTC: Papillary thyroid cancer
qRT-PCR: Quantitative real-time polymerase chain reaction
RAI: Radioiodine
RAI: Radioactive iodine therapy
ROC: Receiver operating characteristic curve
TAM: Tumor-associated macrophages
TCGA: The Cancer Genome Atlas
TILs: Tumor-infiltrating lymphocytes
TILs: Tumor-infiltrating lymphocytes
TISIDB: Tumor-immune system interaction database
TME: Tumor microenvironment
Treg: Regulatory T cells
