# Supplementary figures and images for: High expression of IL4I1 is correlated with poor prognosis and immune infiltration in thyroid cancer
Source: BMC Endocr Disord. 2023 Jul 11;23:148. doi: 10.1186/s12902-023-01407-1 (PMC10334563; doi:10.1186/s12902-023-01407-1)

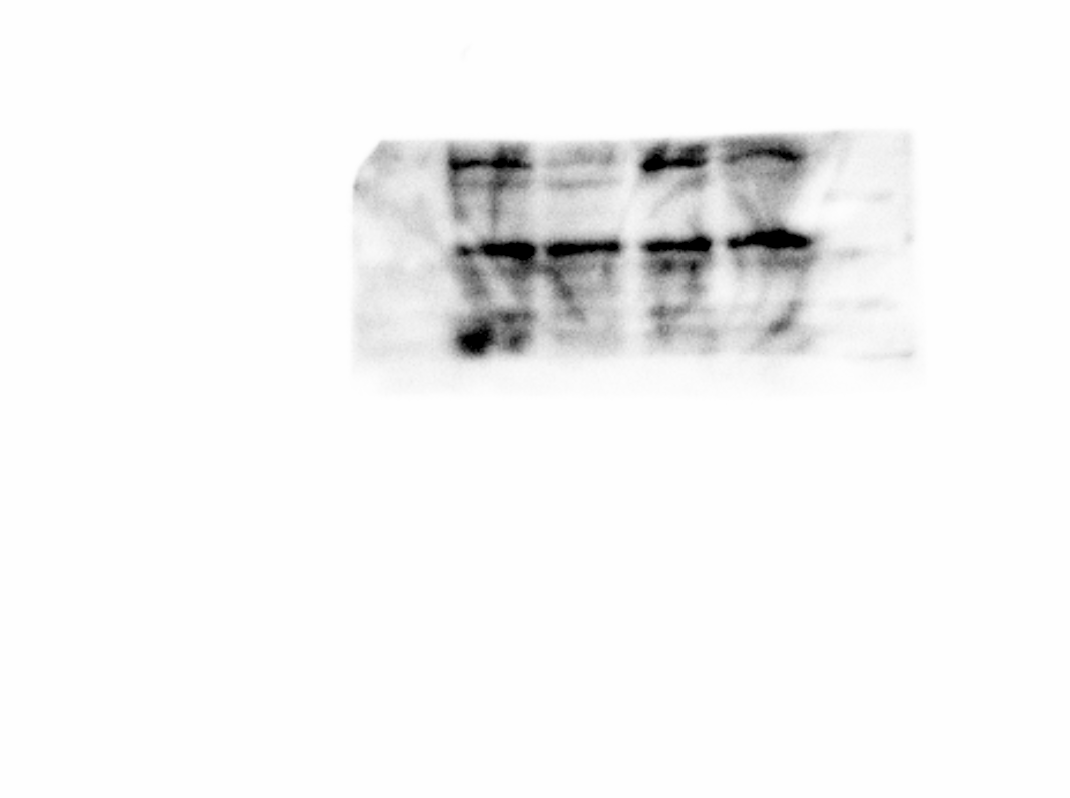

Supplement: Supplementary file 1 — Additional file 1. [file 12902_2023_1407_MOESM1_ESM.tif]

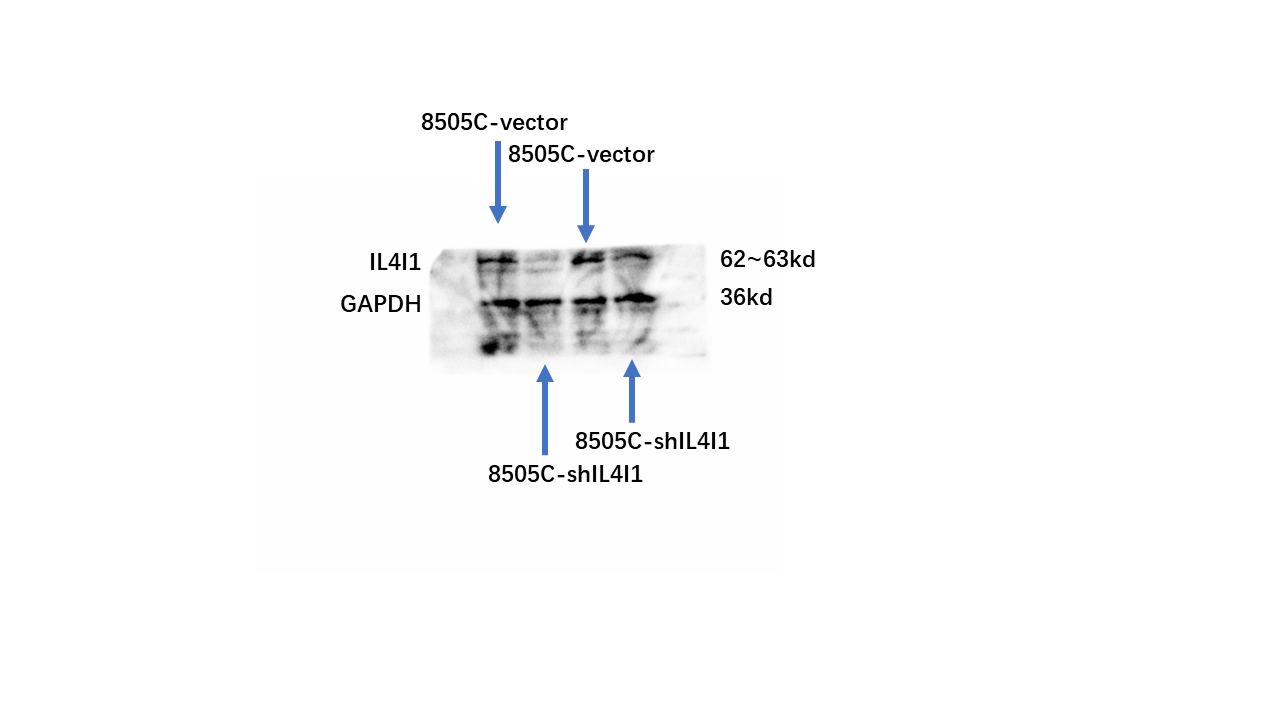

Supplement: Supplementary file 2 — Additional file 2. [file 12902_2023_1407_MOESM2_ESM.tif]
